# Placental extract suppresses the formation of fibrotic deposits by tumor necrosis factor alpha and transforming growth factor beta-induced epithelial–mesenchymal transition in ARPE-19 cells

**DOI:** 10.1186/s13104-021-05824-0

**Published:** 2021-11-02

**Authors:** Kyoko Igarashi, Koji Sugimoto, Eiichi Hirano

**Affiliations:** Research Institute, Japan Bio Products Co., Ltd., 1-1 Kurume Research Center bldg. 2F, Hyakunenkoen, Kurume, 839-0864 Japan

**Keywords:** Placental extract, Bioactivity, Epithelial–mesenchymal transition, Fibrosis, ARPE-19, Proliferative vitreoretinopathy

## Abstract

**Objective:**

Epithelial–mesenchymal transition (EMT) is involved in the development of proliferative vitreoretinopathy (PVR) and subsequent fibrosis. Previously, we demonstrated that placental extract ameliorates fibrosis in a mouse model of non-alcoholic steatohepatitis. In this study, we evaluated whether placental extract influences EMT and fibrosis through cytokine-induced EMT in the retinal pigment epithelial cells, in vitro.

**Results:**

Placental extract did not inhibit EMT, but it suppressed excessive mesenchymal reactions and the subsequent fibrosis. These results suggest that placental extract effectively ameliorates EMT-associated fibrosis in PVR. This beneficial effect could be partially attributed to the suppression of excessive mesenchymal reactions.

**Supplementary Information:**

The online version contains supplementary material available at 10.1186/s13104-021-05824-0.

## Introduction

Proliferative vitreoretinopathy (PVR) is a complication with very poor visual outcomes, following the surgery for retinal detachment and ocular injury [1, 2]. PVR is characterized by the formation of a proliferative membrane in which cells migrate, and then proliferate under the retina or on the retina; this is triggered by ocular injury or retinal tear [3–5]. The cells involved in proliferation are the retinal pigment epithelial cells (RPE), astrocytes, fibroblasts, myofibroblasts, and macrophages. The main cellular components of PVR membranes are RPE cells. The RPE cells are non-proliferating, monolayered, non-motile, and polar, under normal conditions. However, RPE cells proliferate and dedifferentiate to form a multilayer structure under pathological conditions, such as retinal detachment [6]. Epithelial-mesenchymal transition (EMT) is characterized by a loss of epithelial cell phenotype and a gain of mesenchymal phenotype, including the enhancement of cell motility and migration. EMT is functionally classified into three subtypes: type I, II, and III. Type I EMT is related to implantation and embryogenesis, type II is associated with inflammation and fibrosis, and type III is closely related to tumor metastasis. Most studies on EMT involve cancers [7]. The major pathogenesis of PVR could be the conversion of RPE cells into mesenchymal cells through EMT [8]. During chronic inflammation, EMT is continuously induced by cytokines, growth factors, and reactive oxygen species [9], and fibrosis is induced by excessive extracellular matrix deposition, indicating that fibrogenesis is caused by EMT [10].

Placental extract has many biological activities, such as stimulation of liver regeneration [11], anti-oxidation [12], anti-apoptosis [9], and the suppression of cardiac hypertrophy [13]. Clinically, placental extract is an injectable made from the components derived from the human placenta; it improves liver function in chronic hepatitis. Oral porcine placental extract ameliorates menopausal symptoms. Placental extract ameliorates fibrosis in a methionine- and choline-deficient diet-induced mouse model of non-alcoholic steatohepatitis [13, 14]. Therefore, we hypothesized that placental extract could attenuate fibrosis caused by EMT. We evaluated whether placental extract influences EMT and fibrosis by inducing EMT in retinal pigment epithelial cells in vitro.

## Main text

### Materials and methods

#### Placental extracts and cytokines

Porcine or equine placental extracts were obtained from Japan Bio Products Co., Ltd. (Tokyo, Japan). Human recombinant tumor necrosis factor α (TNF-α) and transforming growth factor β2 (TGF-β2) were obtained from Peprotech.

#### Cell culture

Human ARPE-19 cells (from ATCC cell line) were cultured in Ham's F12 media mixed with DMEM (1:1) and supplemented with 10% fetal bovine serum, 2 mM L-glutamine, 100 units/mL penicillin, and 100 μg/mL streptomycin, at 37 °C in humidified air containing 5% CO_2_.

#### Cell viability assay

ARPE-19 cells were placed on 96-well plates at a density of 1.0 × 10^4^ cells/well. After 24 h, cells were washed with culture media once, and then treated with a serial dose of equine and porcine placental extract for 96 h. The cytotoxicity of equine and porcine placental extract on ARPE-19 cells was evaluated using the sulforhodamine B (SRB) assay. The cells were fixed with 50% Trichloroacetic acid for 60 min in fridge, washed with tap water, air-dried at room temperature for 60 min, and stained with 0.4% SRB solution (Sigma) at room temperature for 25 min. After neutralization with 10 mM Tris base solution, and then were measured the absorbance at 565 nm in a microplate reader. This assay was independently repeated on three times.

#### EMT-associated fibrotic deposits (EAFD) assay

EMT-associated fibrotic deposits (EAFD) assay was performed as described previously [15]. ARPE-19 cells were placed on 12-well plates at a density of 5.0 × 10^4^ cells/well. After 72 h, cells were washed with culture media without serum twice, and then cultured for 96 h without serum in the presence of TNF-α (10 ng/mL), TGF-β2 (5 ng/mL), or a serial dose of placental extract. The cells were fixed with methanol for 5 min, air-dried at room temperature for 5 min, and stained with Giemsa solution (Merck) for 15 min. The number of EAFDs was counted using a light microscope and normalized using Photoshop. This assay was independently repeated on three times.

#### Quantitative real-time PCR (qPCR)

Culture and drug treatment of ARPE-19 cells were as same as above “EMT-associated Fibrotic Deposits (EAFD) Assay” section. Total RNA was extracted from ARPE-19 cells using TRI Reagent (TR118, MRC, OH, USA) and cDNA was synthesized using the PrimeScript RT reagent Kit with gDNA Eraser (Takara Bio Inc., Japan). qPCR was performed as previously described [16]. The primer sequences are listed in Additional file 1: Table S1. The data were analyzed using the 2^−ΔΔ^C_T_ method and normalized to the levels of human *TATA-box binding protein* (*Tbp*) mRNA, which was used as an endogenous control. This assay was independently repeated on three times.

#### Statistical analysis

All values are expressed as mean ± SD of at least two independent experiments. One-way ANOVA with *post-hoc* Scheffe’s test was used to determine the significance of differences. Statistical significance was set at p < 0.05.

## Results

### Influence of placental extract on the ARPE-19 cell viability

**Fig. 1:**
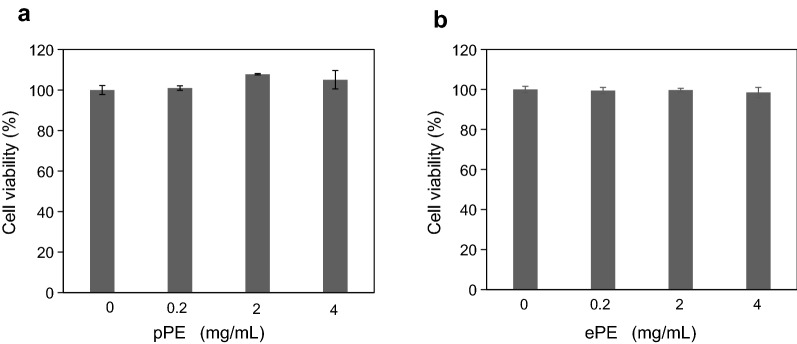
Effect of placental extract on the viability of ARPE-19 cells. Cell viability was measured using the SRB assay. ARPE-19 cells were incubated with varying concentrations (0.4, 2.0, and 4.0 mg/mL) of placental extract, for 96 h. **a** pPE and **b** ePE treatments. Data are expressed as mean ± SD of three independent experiments performed in triplicate

The cell viability following 96 h of treatment with pPE and ePE in ARPE-19 cells was determined using the SRB assay, and the results are shown in Fig. 1. Cell viability was 100.9% ± 1.1%, 107.8% ± 0.4%, and 105.0% ± 4.5% following treatment with 0.2, 2.0, and 4.0 mg/mL of pPE, respectively. In the case of ePE treatment, cell viability was 99.4% ± 1.6%, 99.7% ± 0.9%, and 98.5% ± 2.5% following treatment with 0.2, 2.0, and 4.0 mg/mL of ePE, respectively. Therefore, there was no significant difference in the cell viability in both pPE and ePE treatments, compared to that in the control.

### Inhibitory effect of placental extract on the formation of EAFD in TNF-α and TGF-β2 treated ARPE-19 cells

**Fig. 2:**
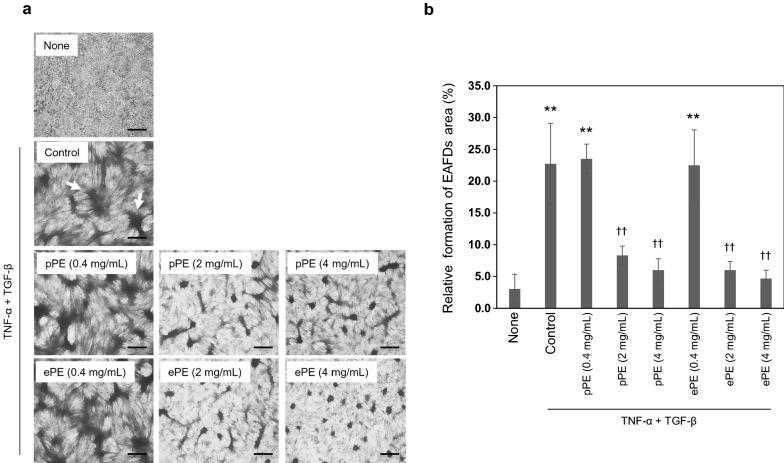
Placental extract inhibited the formation of EAFDs by TNF-α and TGF-β2 in ARPE-19 cells. **a** The formation of EAFDs was detected using Giemsa staining and the cells were counted through microscopic observation. Data are expressed as mean ± SD of at least four different fields, in each of three independent experiments. Arrows indicate EMT-induced EAFDs. Scale bars, 500 μm. **b** Quantification of the EAFD areas. Representative data are shown. ^**^p < 0.01 vs. None. ^††^p < 0.01 vs. Control

It has been reported that the combinative effect of tumor necrosis factor α (TNF-α) with transforming growth factor β2 (TGF-β2) induce EMT in ARPE-19 cells, resulting in the formation of cellular aggregates, which are EMT-associated fibrotic deposits (EAFD) [15, 20]. TNF-α- and TGF-β2 induced EMT in ARPE-19 cells is an initiating event in many fibrotic processes that occur during the pathogenesis of PVR [8]. We studied the effects of placental extract on the formation of EAFD in TNF-α-and TGF-β2 treated ARPE-19 cells using Giemsa staining of EAFD. Treatment with low dose of pPE (0.4 mg/mL) or ePE (0.4 mg/mL) did not influence the formation of EAFD in TNF-α and TGF-β2 treated ARPE-19 cells (Fig. 2a). However, high dose (at 2 and 4 mg/mL) treatment remarkably inhibited the formation of EAFD (Fig. 2a). The area of EAFDs significantly decreased in the placental extract-treated group, compared to that in the control (2 mg/mL pPE; 23 ± 6.4 vs. 8.3 ± 1.5, p < 0.01; 4 mg/mL pPE; 23 ± 6.4 vs. 6.0 ± 1.8, p < 0.01; 2 mg/mL ePE; 23 ± 6.4 vs. 6.0 ± 1.4, p < 0.01; 4 mg/mL ePE; 23 ± 6.4 vs. 4.7 ± 1.3, p < 0.01) (Fig. 2b).

### Effect of equine placental extract on the expression EMT marker and development of PVR -related genes in TNF-α and TGF-β2 treated ARPE-19 cells

**Fig. 3:**
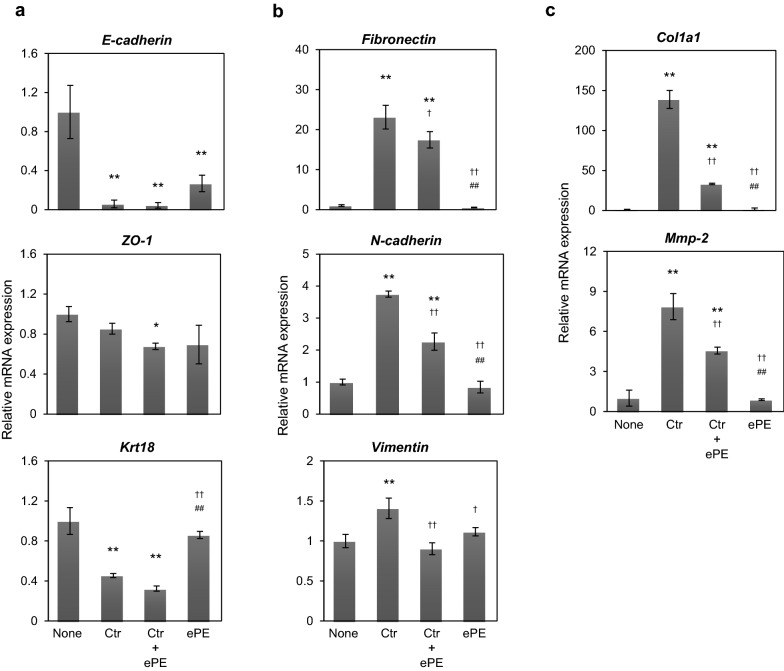
Effect of equine placental extract on the expression of EMT marker and development of PVR -related genes in TNF-α and TGF-β2 treated ARPE-19 cells. The expression levels of the epithelial and mesenchymal phenotype marker genes were determined using qPCR analysis. Cells were treated with TNF-α and TGF-β2 in the presence or absence (control group) of 4 mg/mL ePE..**a** epithelial marker genes (*E-cadherin*, *ZO-1*, *Krt18*), **b** mesenchymal genes (*fibronectin*, *N-cadherin*, and *vimentin*), **c** development of PVR-related genes (*Col1a1* and *Mmp-2*). The results are presented as mean ± SD (n = 3), for three independent experiments. *p < 0.05, **p < 0.01 versus corresponding no treatment (none group), ^†^p < 0.05, ^††^p < 0.01 versus corresponding treatment with TNF-α and TGF-β2 in the absence of ePE. ^##^p < 0.01, versus corresponding treatment with TNF-α and TGF-β2 in the presence of ePE

To evaluate the effects of ePE on the induction of the EMT by TNF-α and TGF-β2 of ARPE-19 cells, we performed qPCR to evaluate the gene expression of *E-cadherin*, *ZO-1*, and *Krt18* (a well-known epithelial markers), and that of *fibronectin*, *N-cadherin*, and *vimentin* (representative mesenchymal markers). TNF-α and TGF-β2 significantly suppressed the expression of the epithelial marker genes (*E-cadherin* and *Krt18*, p < 0.01; *ZO-1*, p < 0.05), compared to that in the none group (neither TNF-α nor TGF-β2-treated group). ePE treatment inhibited the expression of *E-cadherin*, *ZO-1*, and *Krt18* in TNF-α and TGF-β2-treated ARPE-19 cells, compared to that in the control group (TNF-α and TGF-β2-treated group) (Fig. 3a). Among the mesenchymal marker genes, the expression of *fibronectin*, *N-cadherin*, and *vimentin* were significantly increased (p < 0.01) by TNF-α and TGF-β2 of in ARPE-19 cells, compared to that in the none group. ePE significantly blocked the increase in the expression of these epithelial marker genes, compared to that in the control group (*N-cadherin* and *vimentin*, p < 0.01; *fibronectin,* p < 0.05) (Fig. 3b). Since various proteases and extracellular matrix (ECM) are known to be involved in the development of PVR [20], we evaluated the expression level of *Col1a1* and *Mmp-2* genes in ARPE-19 cells. TNF-α and TGF-β2 significantly increased the expression of *Col1a1* and *Mmp-2* (*Col1a1* and *Mmp-2*, p < 0.01), compared to that in the none group; while, ePE significantly blocked the upregulation of these genes, compared to that in the control group (*Col1a1* and *Mmp-2*, p < 0.01) (Fig. 3c).

## Discussion

EMT-inhibiting agents are derived from naturally occurring products such as resveratrol, quercetin, curcumin, and epigallocatechin gallate [17–19]. However, they have been mentioned little of the EMT-associated Fibrotic Deposits, which is EMT-associated fibrosis by TNF-α and TGF-β2-treatment. There are synthetic chemicals that inhibit both EMT and EMT-associated fibrotic deposits in vitro; these include the AMPK activator, 5-aminoimidazole-4-carboxamide ribonucleotide (AICAR); an antiallergy dug, N-[3′,4′-dimethoxycinnamoyl]-anthranilic acid (Tranilast), which is a specific inhibitor of TGF-β receptor I (SB43152); and a hyaluronic acid synthase inhibitor, 4-methylumbelliferone (4-MU) [15, 20, 21]. These chemicals inhibit the formation of EMT-associated fibrotic deposits; while, ePE prevented the growth of EMT-associated fibrotic deposits, suggesting that ePE differs in its mechanism of action from these chemicals.

ePE did not inhibit EMT; however, it prevented excessive mesenchymal reactions and the subsequent fibrosis. However, the suppression of excessive mesenchymal reactions in EMT needs further elucidation. EMT is closely related to tumor metastasis; and therefore, most studies on EMT involve cancers; the therapeutic strategies are mostly focused on the prevention of EMT. Excessive mesenchymal reactions play a key role in the progression of neurofibromas [22]. ePE has a novel mode of action, which leads to the development of new therapeutic strategies, such as combination therapy with ePE and EMT inhibitors. ePE is a novel candidate for the treatment of EMT-associated fibrosis. In addition, the suppressive effect of the excessive mesenchymal reaction could be beneficial in cancer therapy, in particular, in inhibiting the activation of mesenchymal-like cells, such as the MDA-MB-157 (human breast cancer line).

In conclusion, we found that ePE inhibited EMT-associated fibrotic deposits in TNF-α-and TGF-β2-treated ARPE-19 cells. ePE partially suppressed the expression of mesenchymal marker genes (*fibronectin*, *N-cadherin*, and *vimentin*) and ECM-related genes (*Col1a1* and *Mmp-2*) in TNF-α-and TGF-β2-treated ARPE-19 cells. Therefore, ePE could be useful for the prevention of excessive mesenchymal reactions and subsequent fibrosis.

## Limitations

This study was limited to in vitro results, and thus there is a need for further in vivo investigation. Further studies are needed to clarify active ingredients in placental extract for preventing excessive mesenchymal reactions and the subsequent fibrosis, and then elucidate the mode of action of them.

## Supplementary Information

**Additional file 1: Table S1.** Primers used for quantitative real-time PCR.

## Data Availability

The datasets used during this study are available from the corresponding author upon reasonable request.

## Abbreviations

Col1A1: Collagen type I alpha 1 chainCtr: control
ECM: Extracellular matrix
EMT: Epithelial–mesenchymal transition
ePE: Equine placental extract
Krt18: Keratin 18
Mmp-2: Matrix metalloproteinase-2
pPE: Porcine placental extract
PVR: Proliferative vitreoretinopathy
Tbp: TATA-binding protein
ZO-1: Zona occludens-1
