# Supplementary material for: Placental extract suppresses the formation of fibrotic deposits by tumor necrosis factor alpha and transforming growth factor beta-induced epithelial–mesenchymal transition in ARPE-19 cells
Source: BMC Res Notes. 2021 Nov 2;14:407. doi: 10.1186/s13104-021-05824-0 (PMC8561846; doi:10.1186/s13104-021-05824-0)
Supplement: Supplementary file 1 — Additional file 1: Table S1. Primers used for quantitative real-time PCR. [file 13104_2021_5824_MOESM1_ESM.docx]

**Supplementary materials**

Table S1.	Primers used for quantitative real-time PCR

| **Gene** | **Sequence (5’ to 3’)** |
| --- | --- |
| *E-cadherin* | Fw: TGGAGGAATTCTTGCTTTGC  Rv: CGCTCTCCTCCGAAGAAAC |
| *ZO-1* | Fw: AGTAAGTCGTCCTGATCCTGAACC  Rv: TCCTTCTGTTAACCACACCACTCC |
| *Krt18* | Fw: CCTACAAGCCCAGATTGCCA  Rv: CCGAGCCAGCTCGTCATATT |
| *Fibronectin* | Fw: GAACTATGATGCCGACCAGAA  Rv: GGTTGTGCAGATTTCCTCGT |
| *N-cadherin* | Fw: GGTGGAGGAGAAGAAGACCAG  Rv: GGCATCAGGCTCCACAGT |
| *Vimentin* | Fw: TTGCAGGAGGAGATGCTTCA  Rv: TTGCGTTCAAGGTCAAGACG |
| *Col1A1* | Fw: GATCAGAGCATTGTGCAATACAG  Rv: CCATAACAGGTGTAAGAGTGTTGAA |
| *Mmp-2* | Fw: CAGGAGGAGAAGGCTGTGTT  Rv: TTAAAGGCGGCATCCACTCG |
| *Tbp* | Fw: TTGGGTTTTCCAGCTAAGTTCT  Rv: CCAGGAAATAACTCTGGCTCA |

**Response to reviewer comments**

Dear Editor-in-Chief,

Thank you for inviting us to submit a revised draft of our manuscript entitled, *Placental extract suppresses the formation of fibrotic deposits by tumor necrosis factor alpha and transforming growth factor beta-induced epithelial-mesenchymal transition in ARPE-19 cells*. We also appreciate the time and effort you and each of the reviewers have dedicated to providing insightful feedback on ways to strengthen our paper. Thus, it is with great pleasure that we resubmit our article for further consideration. We have incorporated changes that reflect the detailed suggestions you have graciously provided. We also hope that our edits and the responses we provide below satisfactorily address all the issues and concerns you and the reviewers have noted.

To facilitate your review of our revisions, the following is a point-by-point response to the questions and comments delivered in your letter dated.

**Editor Comments**:

- *As per the reviewers comments, please ensure that all methods are reported with enough detail to allow the repeatability of the experiments.*

We appreciated this comment on this point. We agree with you. We have added more details in the following sections.

Materials and methods section, in ***1-3. Cell viability assay***: page 6, line 17-25; ***1-4. EMT-associated Fibrotic Deposits (EAFD) Assay***: page 7, line 2-3 and 8; **1-5. *Quantitative real-time PCR (qPCR)***: page 7, line 11-12 and 17-18, indicated by a yellow highlight.

- *When presenting your results, please use a consistent number of decimal places throughout and specifically ensure that the numbers and their errors are reported to the same level of accuracy.*

We thank for this comment. We have been corrected in accordance with this comment in the Results sections, ***2-1. Influence of placental extract on the ARPE-19 cell viability***: in page. 8, line 3 and 5, indicated by a yellow highlight.

- *We noted that in figure 2 a scale bar was only added to one of the microscopy images. Please ensure scale bars are present in all panels.*

In accordance with this comment, we have presented scale bars in all panels in Figure 2a.

- *Please revise you availability of data and materials statement to detail how the data on which the conclusions rely can be accessed by the readers post-publication.*

We agree with you. We have changed this to “The datasets used during this study are available from the corresponding author upon reasonable request” described in the Declarations section, **Availability of data and materials**: in page11, line 26-27, indicated by a yellow highlight.

**Reviewer 1**: *In this study the effect of porcine and equine placental extracts have been studied on epithelial-mesenchymal transition and fibrosis markers in human ARPE-19 cell cultures. While the idea is interesting, Materials and Methods are severely lacking of details (manufacturers, cell numbers, incubation conditions, washes etc.). Please, remember that the study should be able to be repeated on the basis of this paper. In addition, cell viability assays should be performed also under the induction of EMT.*

We appreciated this comment on this point. We agree with you. We have added more details in the following sections.

Materials and methods section, in ***1-3. Cell viability assay***: page 6, line 17-25; ***1-4. EMT-associated Fibrotic Deposits (EAFD) Assay***: page 7, line 2-3 and 8; **1-5. *Quantitative real-time PCR (qPCR)***: page 7, line 11-12 and 17-18, indicated by a yellow highlight.

We agree that additional information on cell viability assays as the reviewer suggested would be valuable. Regrettably, however, because of technical problem, we are unable to do the experimentation. In this study, we observed that ARPE-19 cells became spindle-shaped and gathered together to form piled up cellular aggregates after treatment in the presence of both TNF-alpha and TGF-beta2, with these aggregates being termed EMT- associated fibrotic deposits (EAFD). These findings are consisted with previous report [1, 2]. It is indicated that EAFD would not a monolayer, but a three-dimensional one. In fact, the observed EAFD shows that the boundaries between cells are ambiguous, indicating that the cells are three-dimensional. In this situation, conventional reagents (SRB and MTT) for detecting cell viability do not allow these reagents to fully penetrate this EAFD. In addition, the penetrance of these reagents into cells is presumed to be that the penetrance of non-EAFD and the penetrance of EAFD differ significantly in both time and space. Therefore, in this situation where non- EAFD and EAFD coexist, we have judged that the conventional method lacks credibility in measuring cell viability. It is necessary a new technique to overcome these problems until now.

1. Takahashi, et al. Tumor necrosis factor-alpha regulates transforming growth factor-beta-dependent epithelial-mesenchymal transition by promoting hyaluronan-CD44-moesin interaction. J Biol Chem. 2010;285:4060-73.
2. Matoba, et al. Suppressive effect of AMP-activated protein kinase on the epithelial-mesenchymal transition in retinal pigment epithelial cells. PLoS One. 2017;12:e0181481.

Again, thank you for giving us the opportunity to strengthen our manuscript with your valuable comments and queries. We have worked hard to incorporate your feedback and hope that these revisions persuade you to accept our submission.

Sincerely,

Eiichi Hirano, Ph.D.
